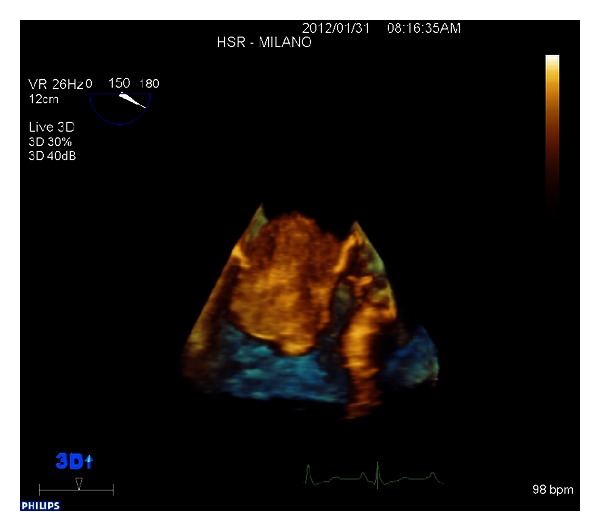# Acute Pulmonary Edema Caused by a Giant Atrial Myxoma

**DOI:** 10.1155/2013/904952

**Published:** 2013-05-16

**Authors:** Andrea Fisicaro, Massimo Slavich, Eustachio Agricola, Claudia Marini, Alberto Margonato

**Affiliations:** Division of Cardiology, San Raffaele University Hospital, Via Olgettina 58, 20100 Milan, Italy

## Abstract

Atrial myxoma is the most common primary cardiac tumor. Its clinical presentation spreads from asymptomatic incidental mass to serious life-threatening cardiovascular complications. We report the case of a 44-year-old man with evening fever and worsening dyspnea in the last weeks, admitted to our hospital for acute pulmonary edema. The cardiac auscultation was very suspicious for mitral valve stenosis, but the echocardiography revealed a huge atrial mass with a diastolic prolapse into mitral valve orifice causing an extremely high transmitral gradient pressure. Awareness of this uncommon acute presentation of atrial myxoma is necessary for timely diagnosis and prompt surgical intervention.

## 1. Introduction

Cardiac tumors are very rare in comparison with other forms of heart disease. Primary cardiac tumors occur 30 times less frequently than cardiac metastases with an autopsy reported prevalence that ranges from 0.001% to 0.2%; about 75% of them are benign [1]. Myxomas are the most common benign cardiac primary tumors, accounting for approximately 40% to 50%; they are more prevalent among women with a mean age of 50 years [2]. About 80% are found in left atrium, where the typical site of attachment is near the fossa ovalis [3]. 

Here, we report the diagnostic evaluation of a giant atrial myxoma in a 44-year-old man that caused acute pulmonary edema as a consequence of mitral valve obstruction.

## 2. Case Presentation

A 44-year-old man was admitted to the Intensive Care Unit for acute pulmonary edema. He referred worsening exertional dyspnea, paroxysmal nocturnal dyspnea, and orthopnea during the last four weeks; he also complained of intermittent low-grade evening fever during the last days. His past medical history was unremarkable, and he denied any common cardiovascular risk factor. On admission to hospital, the patient presented a blood pressure of 110/60 mmHg. The chest examination found diffuse pulmonary crackles; the cardiac auscultation revealed a suspected opening snap and a diastolic murmur with presystolic accentuation that seems to vary with changes in position. The ECG was normal. Because of the high suspicion for mitral valve stenosis, transthoracic (TTE) and then transesophageal echocardiography (TEE) were performed.

**Figure 1:**
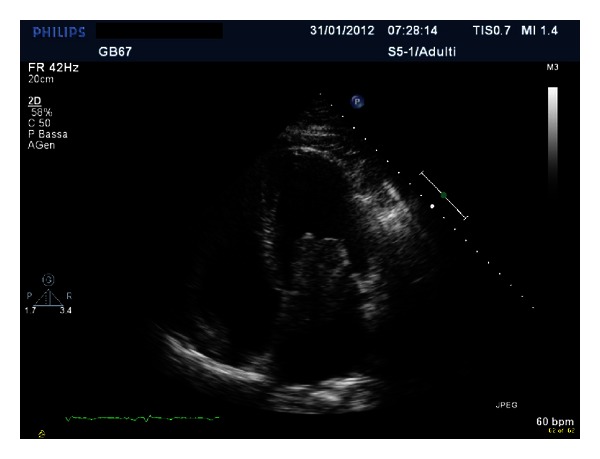


**Figure 2:**
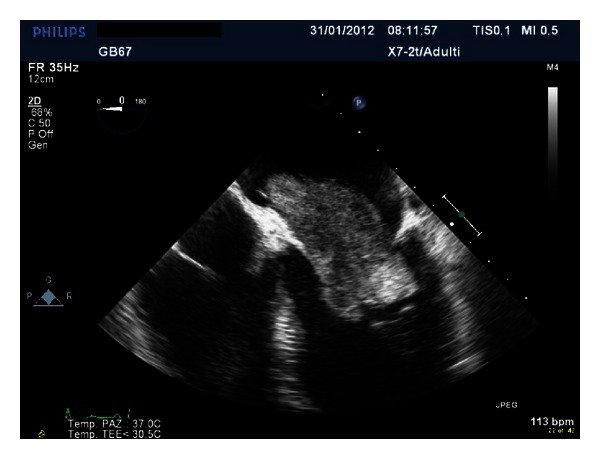


**Figure 3:**
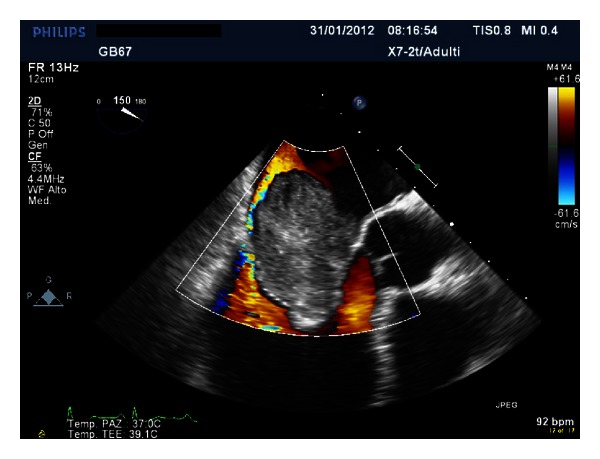


**Figure 4:**
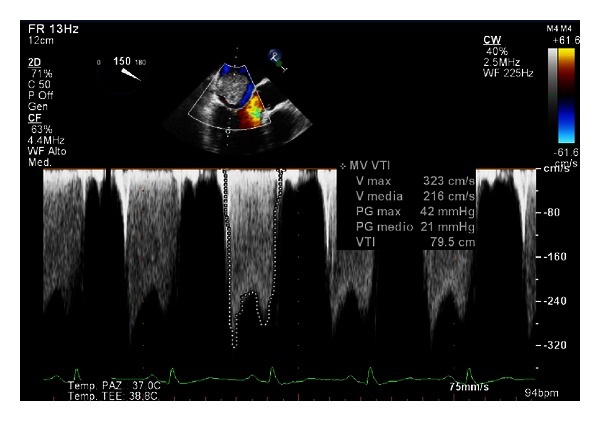


**Figure 5:**
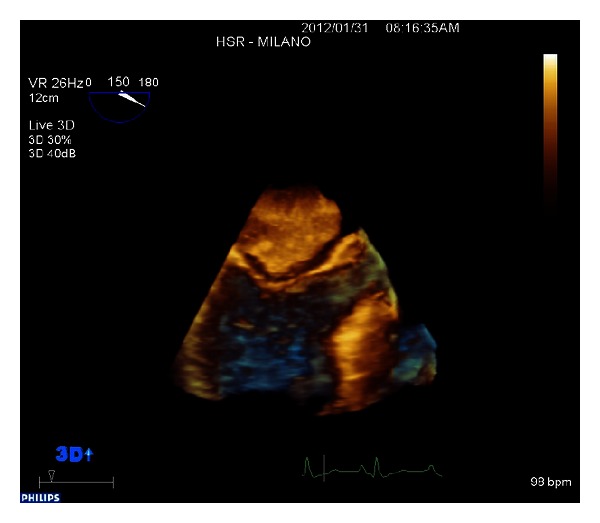


A giant floppy mass (64 × 37 mm) implanted on the left side of the interatrial septum with diastolic movement through the mitral valve into the left ventricle was detected (Figures 1 and 2; see supplementary material available online at http://dx.doi.org/10.1155/2013/904952). The colour Doppler revealed high turbulence due to a diastolic transmitral flow obstruction (Figure 3), and the continuous-wave Doppler confirmed the obstruction with an extremely high transmitral gradient pressure (medium 21 mmHg; maximum 42 mmHg) (Figure 4). Three-dimensional TEE was performed in order to properly evaluate the mass and its relationship with the heart structures (Figures 5 and 6). 

The left side interatrial septum localization near the fossa ovalis and the echogenicity were highly suggestive for an atrial myxoma. The patient received massive doses of diuretic therapy initially and underwent successful surgical mass excision two days later. The mass (70 × 41 mm) was solid and smooth in appearance, attached via a pedunculated base. The surface predominant colour was red due to multiple and diffused areas of haemorrhage.

Histologically, there were cords and syncytia of stellate shaped cells with abundant eosinophilic cytoplasm, within glycosaminoglycan-rich myxoid stroma. These cells formed rings around the small multiple vessels. There were also extravasated red cells and multiple foci of recent and old hemorrhage with hemosiderin deposition. These histopathologic features were consistent with the diagnosis of myxoma.

After 3 months, the patient is in good general condition, and the control TTE was negative.

## 3. Discussion

Myxomas are the most common primary benign cardiac tumors. Although they can be found incidentally in asymptomatic patients, myxomas often present with very nonspecific signs and symptoms. However, they may cause life-threatening cardiac symptoms thus necessitating emergency surgery. 

Myxomas can provide symptoms depending on their location and their relationship with the cardiac structures. Tumors of the left atrium, especially if mobile and of great dimensions, may cause obstruction of atrioventricular blood flow, resulting in symptoms similar to mitral stenosis, such as exertional dyspnea, paroxysmal nocturnal dyspnea, orthopnea, fatigue, and syncope. In this case, the cardiac auscultation may also mimic mitral valve stenosis. In fact a widely split and loud S1 can be found, caused by the late closure of the mitral valve when ventricular-atrial pressure crossover occurs at a higher pressure. The tumor plop of myxoma, a protodiastolic murmur heard 80 to 150 ms after the second heart sound, may be mistaken for an opening snap, and tumor obstruction of the valve leads to a diastolic murmur. Finally a presystolic crescendo murmur may also be present, beginning during ventricular systole as the tumor moves through the mitral valve. However, typically in this condition the auscultatory findings are characteristically variable with changes in position and from examination to examination [4].

Myxomas are also associated with a variety of constitutional manifestation such as fever, malaise, and weight loss, likely attributable to the constitutive production of inflammatory cytokines by the tumor [5].

Finally patients may complain of symptoms due to embolic phenomena [6], such as transient ischemic attack, stroke, myocardial, or visceral infarction.

The peculiarity of our finding consists of the haemodynamic relevance of the mass which mimicked severe mitral valve stenosis leading to acute pulmonary edema, a serious and life-threatening condition. Because of this important hemodynamic impact and the huge dimensions of the mass we decided to define this myxoma as giant.

The opening snap present on admission was actually the tumor plop related to the prolapse of the myxoma across the mitral valve. The worsening symptoms of the last weeks were presumably due to the rapidly growing myxoma [7], which progressively led to a significant gradient across the valve, which in turn led to acute pulmonary edema. 

At the present time, there is no clear consensus regarding the followup of patients undergone to myxoma abscission. Notably, the tumor and atrial septum resection, with or without patch application, when properly performed, is a definitive intervention. In fact, the risk of myxoma recurrence is low and it usually happens within the first months/years. Therefore, it is in this period that echocardiographic monitoring should be performed more strictly in order to early detect an eventual mass recurrence.

In our experience the TEE does not provide additional information compared to the TTE, which appears enough for the followup. However, TEE could be useful in case of poor acoustic window, in particular early after surgery, when TTE is not conclusive and does not allow the correct visualization of the atrial septum.

## Supplementary Material

Transesophageal echocardiography, midesophageal four-chamber view. Huge solid and smooth left atrial mass with diastolic prolapse into the left ventricle causing nearly total mitral valve obstruction and mimicking mitral valve stenosis pathophysiology.

## Figures and Tables

**Figure 6:**